# Supplementary material for: Discordant attributes of structural and functional brain connectivity in a two-layer multiplex network
Source: Sci Rep. 2019 Feb 27;9:2885. doi: 10.1038/s41598-019-39243-w (PMC6393555; doi:10.1038/s41598-019-39243-w)
Supplement: Supplementary file 1 — Supplementary Material [file 41598_2019_39243_MOESM1_ESM.pdf]

Discordant attributes of structural and functional brain connectivity in a two-layer multiplex network

Sol Lim*[1,6], Filippo Radicchi†[2], Martijn P van den Heuvel‡[3,4] and Olaf Sporns§[1,5]

[1]Department of Psychological and Brain Sciences, Indiana University, Bloomington, IN 47405, USA

[2]Center for Complex Networks and Systems Research, School of Informatics, Computing and Engineering, Indiana University, Bloomington, IN 47408, USA

[3]Connectome Lab, Department of Neuroscience, Section Complex Traits Genetics, Center for Neurogenomics and Cognitive Research, Amsterdam Neuroscience, VU Amsterdam, Amsterdam, 1081 HV, The Netherlands

[4]Department of Clinical Genetics, UMC Amsterdam, Amsterdam Neuroscience, Amsterdam, 1081 HV, The Netherlands

[5]Network Science Institute, Indiana University, Bloomington, IN 47408, USA

[6]Brain Mapping Unit, Department of Psychiatry, Cambridge University, Cambridge, CB2 3EB, United Kingdom

*cogscisol@gmail.com;sl904@cam.ac.uk;limsol@indiana.edu
†f.radicchi@gmail.com
‡martijn.vanden.heuvel@vu.nl
§osporns@indiana.edu

Supplementary Material

S1. Yeo's canonical 7 functional subnetworks.

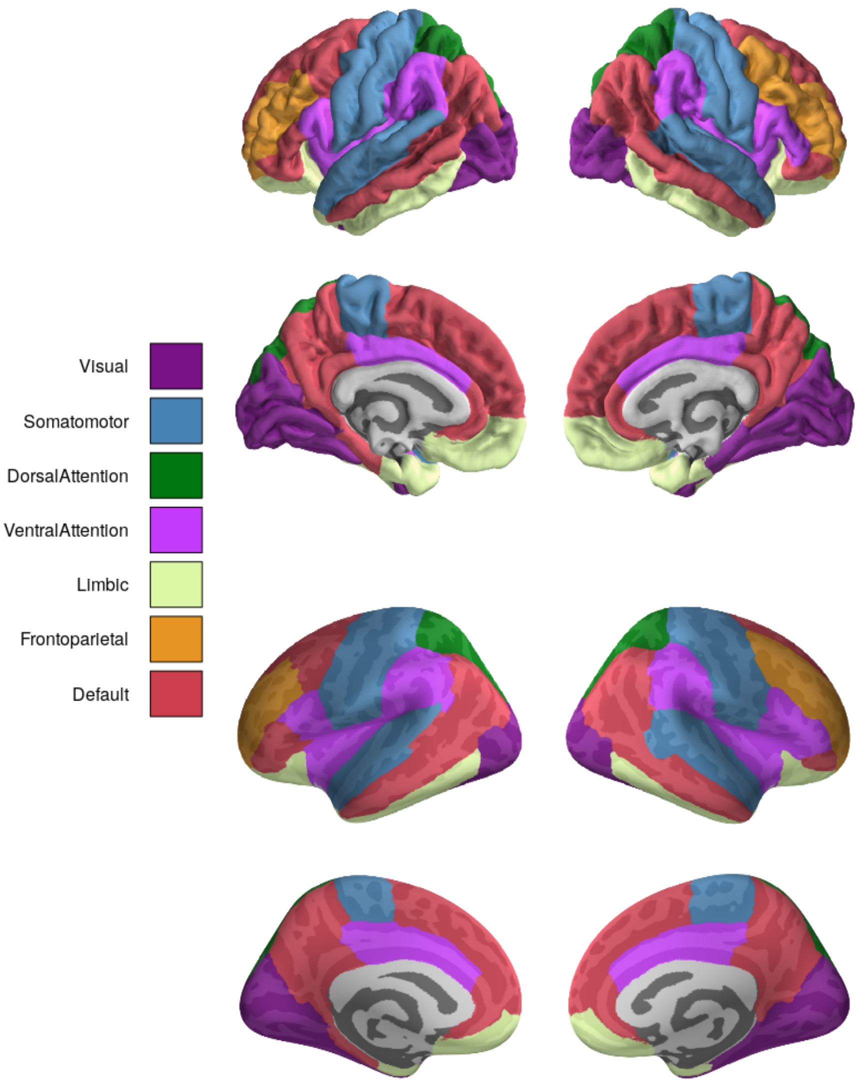

Figure S1. (Left) Yeo's 7 subnetworks on the pial surface, (Right) Yeo's 7 subnetworks on the inflated surface. The colors are matched with the original colors in the paper [1].

S2. Network size and within-layer node strength assortativity within FC and SC and between-layer assortativity between FC and SC.

We have explored the impact of the number of regions on the results since degree disassortative networks have shown a decreasing degree disassortativity as the network size increases [2], by using 68 cortical regions (Desikan-Killiany Atlas), Lausanne 114 cortical regions and Lausanne 219 cortical regions [3]. As can be seen in the following figure (Figure S2) the size of the network affects node strength assortativity coefficients; however, the disassortativity of FC remained the same and the contrasting assortativity results between FC and SC were retained as well in three different network sizes. The decreasing disassortativity when the network size increases mainly is attributed the dispersion of the degree distribution as the network size increases; however, the strength distribution of FC does not show a broader distribution. In contrast, the correlation coefficient between FC and SC

layers did decrease as the network size increased.

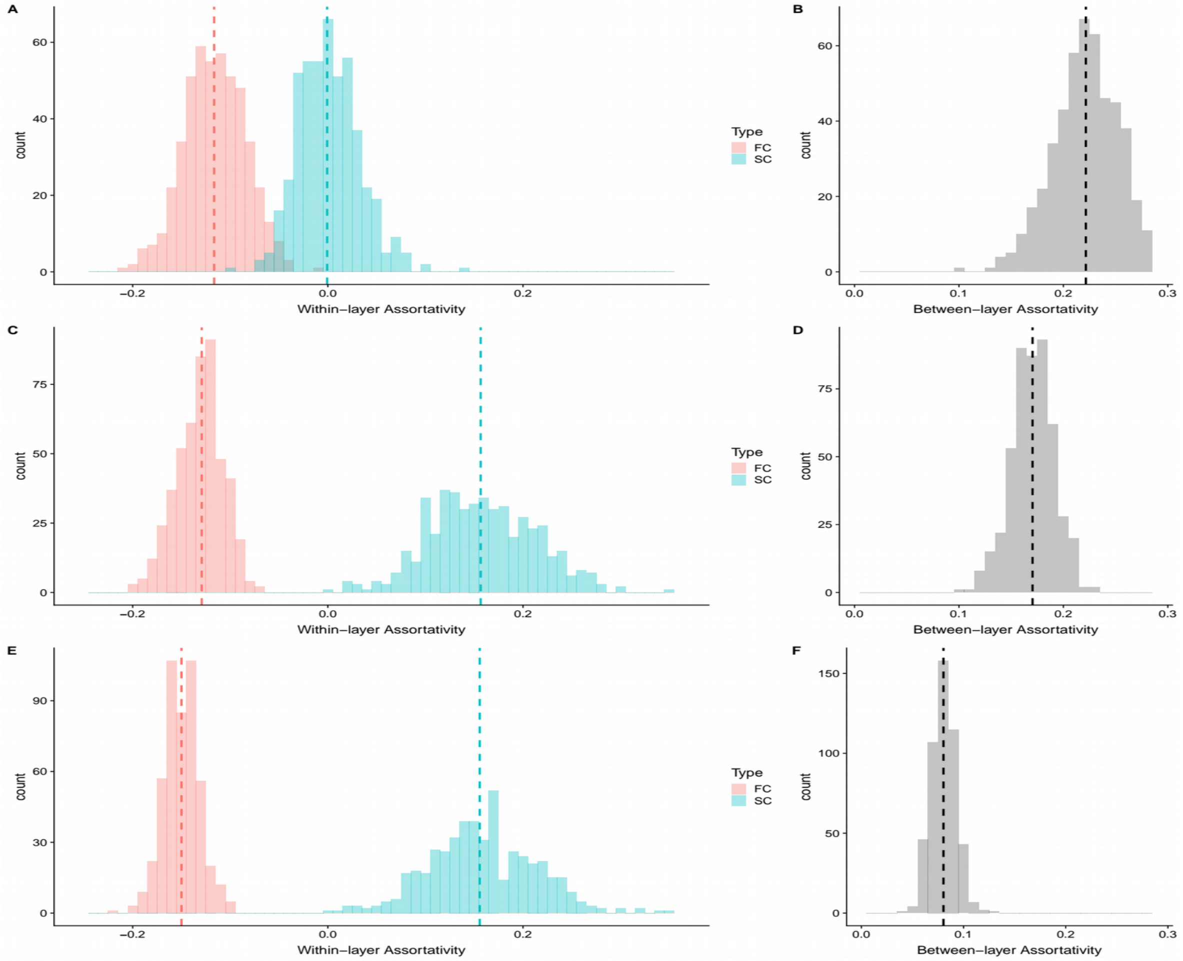

Figure S2. (Left) A, C, E: Within-layer assortativity: histograms (tallying numbers of individual subjects) of the strength assortativity within the functional network (FC) and the structural network (SC), respectively. Red: FC, Blue: SC. (Right) B, D, C: Between-layer assortativity between FC and SC. (First row) A and B: Desikan-Killiany atlas 68 ROIs, (Second row) C and D: Lausanne 114 cortical regions and (Third row) E and F: Lausanne 219 cortical regions, which are subdivision of Desikan-Killiany atlas [3].

S3. The range of distribution depending on the network size

Assortativity estimation based on Pearson's correlation coefficient is shown to be influenced by the distribution of the degrees of the nodes in the network [2]. Thus, we examined the potential bias based on the difference in the ranges between SC and FC using three different network sizes. In, particular, we investigated if the disassortativity of FC was from a broader strength distribution of nodes in FC. In fact, the strength distribution of SC is much broader than that of FC. Hence, the smaller assortativity value (or disassortativity) for FC is not attributable to a wider range of the degree distribution of FC. The following figure shows the node strength distribution ranges for all subjects. The range of the node strength distribution of an individual was calculated by the difference between the maximum node strength value and the minimum node strength value for each individual. The three histograms show all 484 participants for different resolutions of parcellations of the brain.

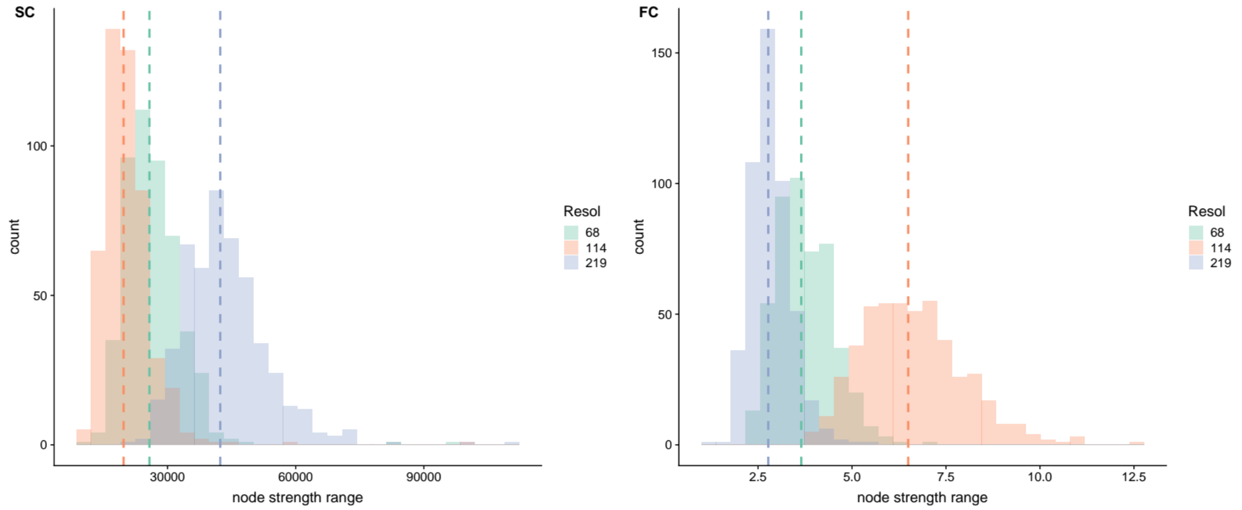

Figure S3. (Left) the range distribution for all 484 subjects in SC in three difference network sizes. (Right) the range distribution for all subjects in FC. Green: 68 ROIs Desikan-Killiany atlas, Red: 114 Lausanne parcellation and Blue: 219 Lausanne parcellation [3].

Of note, here the range of nodes strength distribution depending on the network size may have been confounded by the fact that the bigger networks (114 ROIs and 219 ROIs) [3] are constructed by subdividing the original Desikan-Killiany 68 parcellation.

S4. Within-layer assortativity of the strength assortativity within the functional network (FC) and the structural network (SC) and between-layer assortativity between FC and SC using Spearman’s rho.

Moreover, we have used Spearman’s rho as recommended in this paper [2] to account for a possible bias due to the strength distribution differences between SC and FC and in different network sizes. Our results in the following figure showed no qualitative differences when we used Spearman’s rho instead of Pearson’s correlation coefficient.

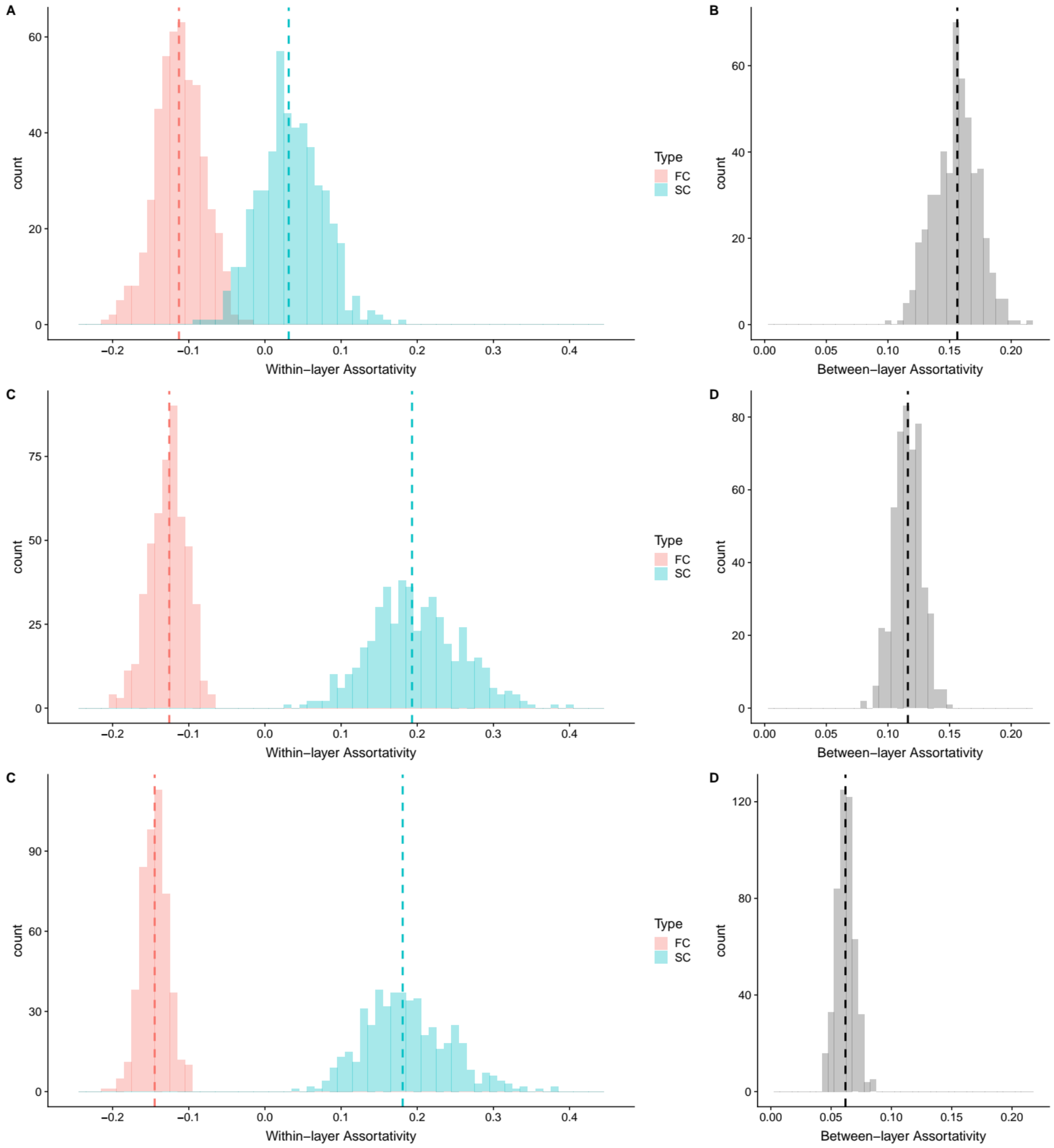

Figure S4. Within- and between-layer node strength assortativity based on Spearman's rho

(Left) A, C, E: Within-layer assortativity: histograms (tallying numbers of individual subjects) of the strength assortativity within the functional network (FC) and the structural network (SC), respectively. Red: FC, Blue: SC. (Right) B, D, C: Between-layer assortativity between FC and SC. (First row) A and B: Desikan-Killiany atlas 68 ROIs, (Second row) C and D: Lausanne 114 cortical regions and (Third row) E and F: Lausanne 219 cortical regions, which are subdivision of Desikan-Killiany atlas [3].

S5. Local assortativity

Local assortativity for each ROI was calculated using [4, 5], which was based on the global assortativity of the entire network. These values change depending on the normalizing constant and relative to the network where they are calculated. For example, the local assortativity values for the same set of nodes would be different when they are estimated them from the subnetworks because the global assortativity for the subnetwork is different from the global assortativity for the entire network.

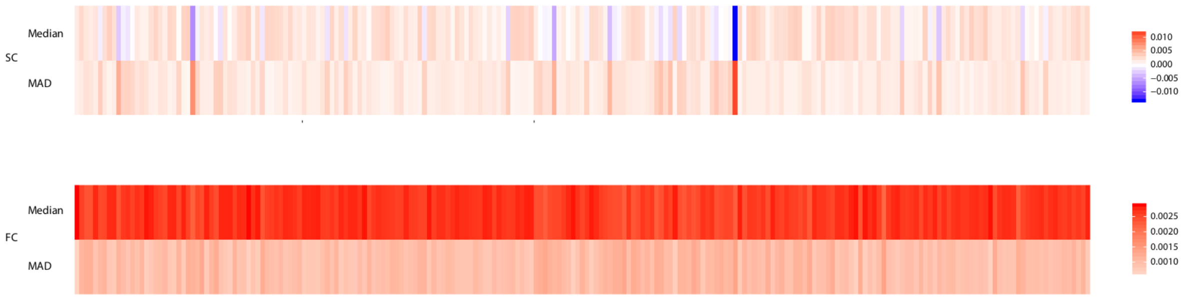

Figure S5. (Top) Local assortativity for structural connectivity. 1st row: the median values of the nodal assortativity of all subjects, 2nd row: the median absolute values of the nodal assortativity. (Bottom) Local assortativity for functional connectivity. 1st row: the median values of the nodal assortativity of all subjects, 2nd row: the median absolute values of the nodal assortativity.

Local assortativity for all subjects for SC and FC

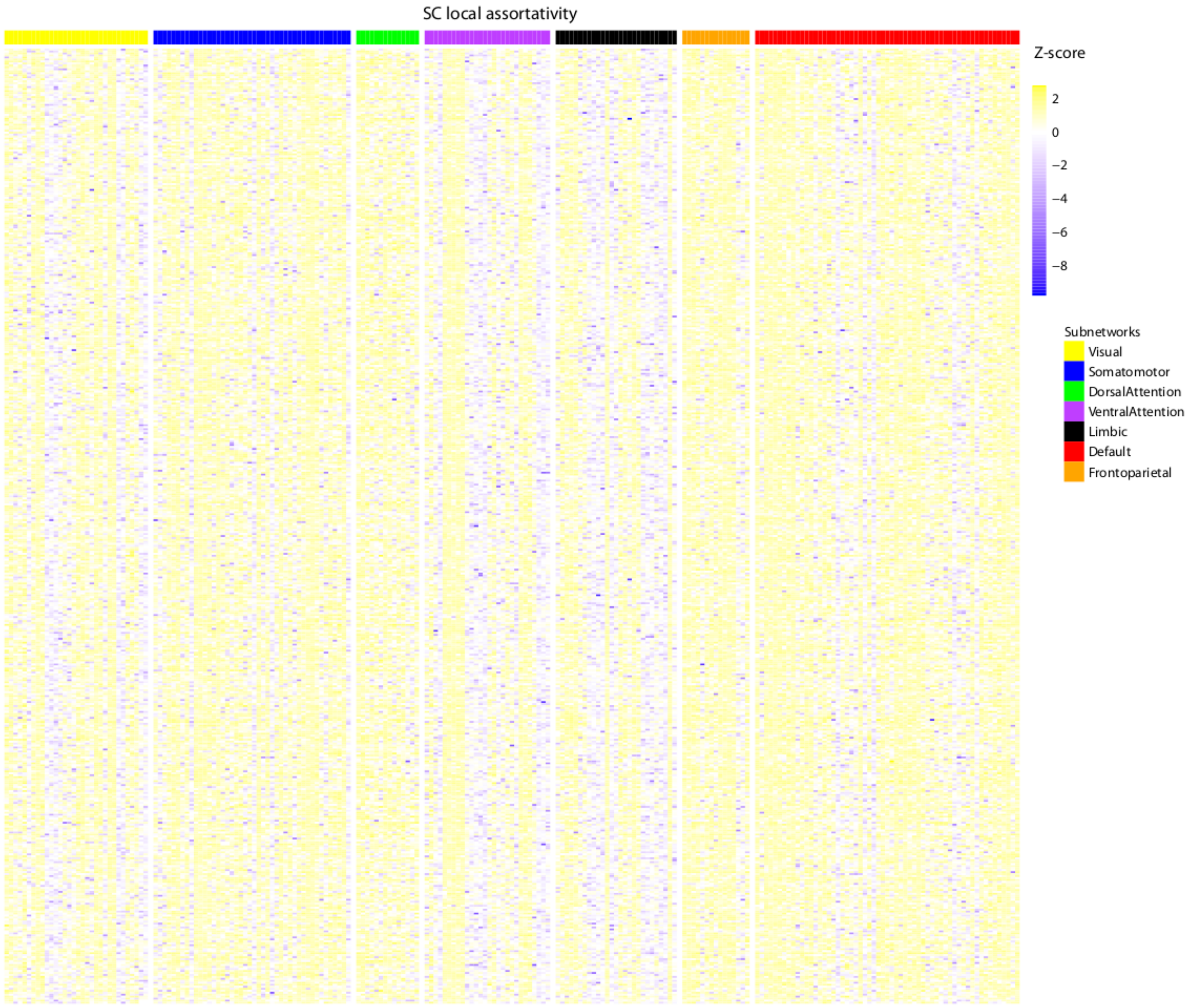

Figure S6.Local assortativity of SC for all subjects. (row) all subjects (column) local assortativity rearranged based on canonical functional 7 networks.

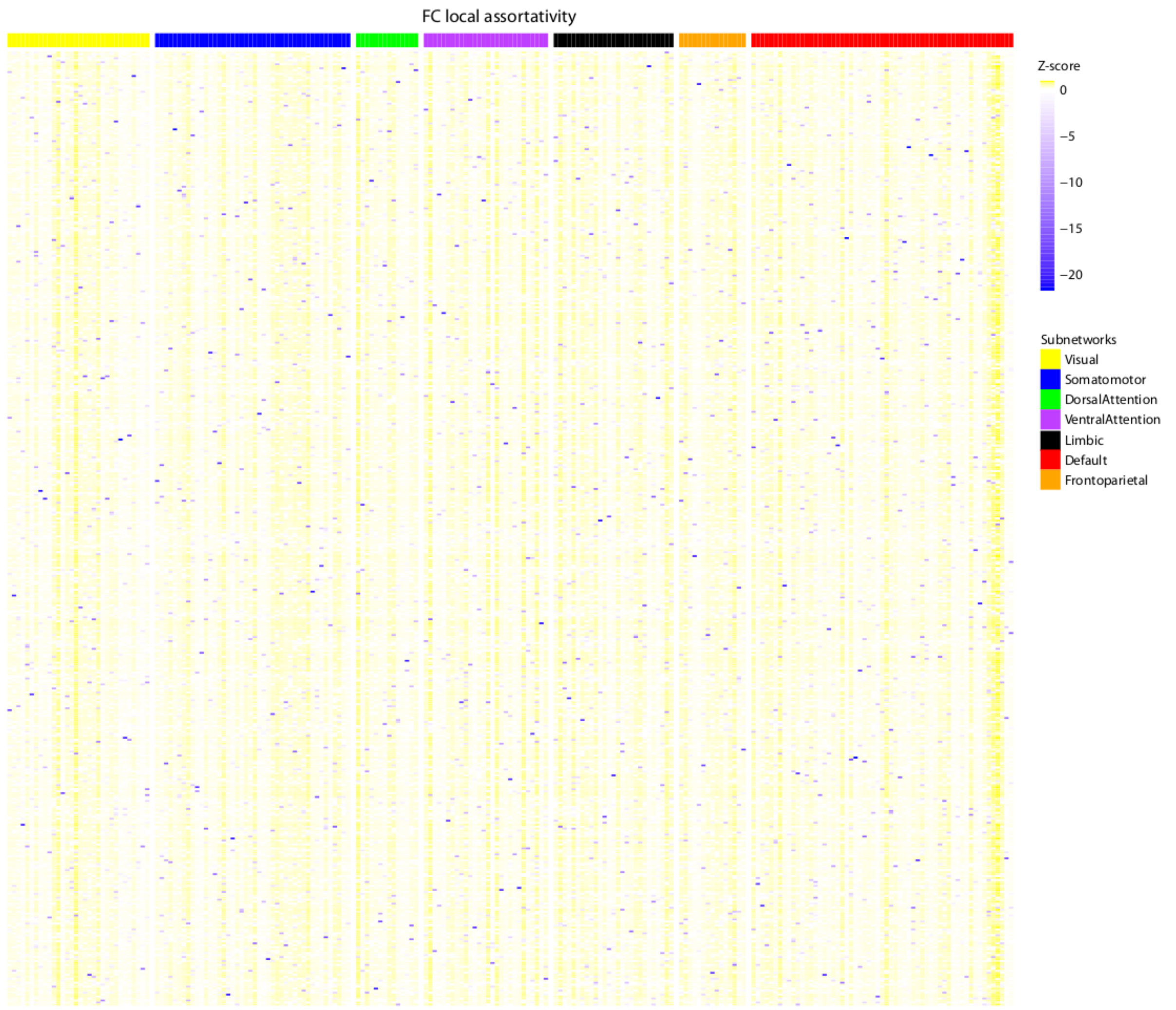

Figure S7.Local assortativity of FC for all subjects. (row) all subjects (column) local assortativity rearranged based on canonical functional 7 networks.

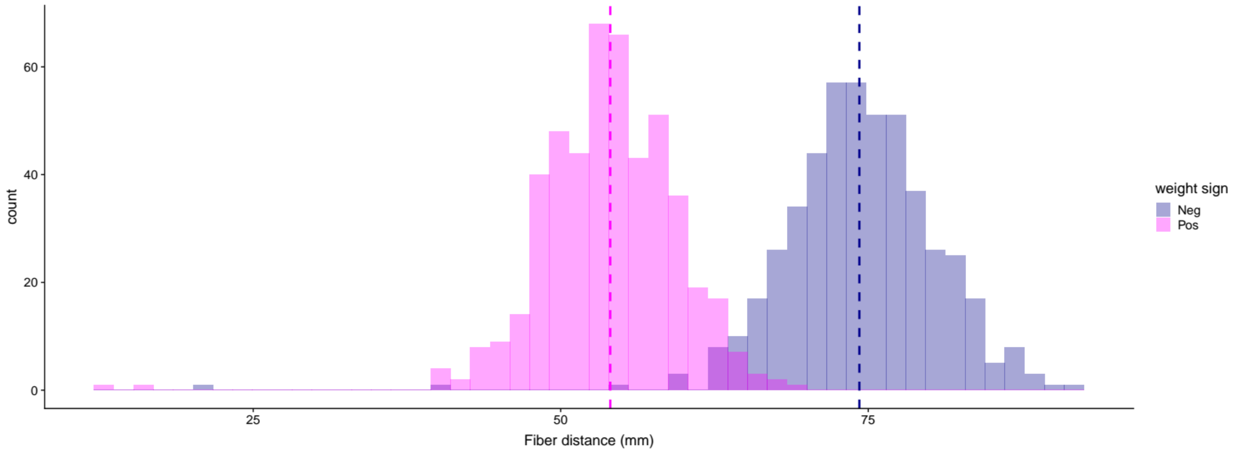

Figure S8. Fiber length histogram of edges with positive and negative weights. X-axis: fiber distance (mm), Y-axis: count, Pink: edges with positive weights, Blue: edges with negative weights

References

[1] Yeo, BT Thomas, et al. "The Organization of the Human Cerebral Cortex Estimated by Functional Connectivity 2." *network* 21 (2011): 22.

[2] Litvak, Nelly, and Remco Van Der Hofstad. "Uncovering disassortativity in large scale-free networks." *Physical Review E* 87.2 (2013): 022801.

[3] Cammoun, Leila, et al. "Mapping the human connectome at multiple scales with diffusion spectrum MRI." *Journal of neuroscience methods* 203.2 (2012): 386-397.

[4] Thedchanamoorthy, Gnana, et al. "Node assortativity in complex networks: An alternative approach." *Procedia Computer Science* 29 (2014): 2449-2461.

[5] Rubinov, Mikail, and Olaf Sporns. "Complex network measures of brain connectivity: uses and interpretations." *Neuroimage* 52.3 (2010): 1059-1069.
